# Associations of sleeping, sedentary and physical activity with phenotypic age acceleration: a cross-sectional isotemporal substitution model

**DOI:** 10.1186/s12877-023-03874-6

**Published:** 2023-03-23

**Authors:** Mengying Han, Jiaxin Fang, Yixin Zhang, Xingxu Song, Lina Jin, Yanan Ma

**Affiliations:** 1 Department of Biostatistics and Epidemiology, School of Public Health, China Medical University, No. 77 Puhe Road, Shenyang North New Area, Liaoning Province 110122 Shenyang, P.R. China; 2 Department of Epidemiology and Biostatistics, School of Public Health, Jilin University, No.1163 Xinmin Street, Changchun, Jilin, 130021 China

**Keywords:** Isotemporal substitution model, Physical activity, Lifestyle, Aging

## Abstract

**Background:**

Physical activity was believed to be associated with reduced aging among adults, while the competing nature of the physical activity and sedentary behavior has mainly been neglected in studies. We aimed to estimate the association of sleeping, sedentary behavior, and physical activity with aging among adults, considering the competing nature between variables of activity status.

**Methods:**

A total of 5288 participants who were 20 years or older from the National Health and Nutrition Examination Survey were involved. The questionnaire was used to collect data regarding sociodemographics (age, sex, ethnicity/race, and education), and lifestyle behaviors (smoking, drinking). The Global Physical Activity Questionnaire was used to measure self-reported time for sedentary behavior, walking/bicycling, and moderate-to-vigorous physical activity (MVPA). The sleeping duration was obtained via interview. Phenotypic age acceleration (PhenoAgeAccel) was calculated as an aging index using nine chemistry biomarkers. Isotemporal substitution models using multivariable linear regression to examine the associations of sleeping, sedentary behavior, and physical activity with PhenoAgeAccel, stratified by MVPA (< 150 min/week, ≥ 150 min/week).

**Results:**

Thirty minutes per day spent on sedentary behavior was positively associated with PhenoAgeAccel (*β* = 0.07, 95% CI: 0.04, 0.11), and 30 min/day spent on leisure-time MVPA was adversely associated with PhenoAgeAccel (*β* =  − 0.55, 95% CI: − 0.73, − 0.38). Replacing 30 min/day sedentary behaviors with 30 min/day of MVPA (*β* = -3.98, 95% CI: -6.22, -1.74) or 30 min/day of walking/bicycling (*β* = -0.89, 95% CI: -1.10, -0.68) was adversely associated with PhenoAgeAccel. Substituting 30 min/day of walking/bicycling for 30 min/day of leisure-time MVPA was positively associated with PhenoAgeAccel (*β* = 3.09, 95% CI: 0.93, 5.25).

**Conclusion:**

Sedentary behavior was positively associated with aging. Replacing sedentary behaviors with walking/bicycling or MVPA was adversely associated with aging among adults.

**Supplementary Information:**

The online version contains supplementary material available at 10.1186/s12877-023-03874-6.

## Impact statement

We demonstrate that this work is novel: For the first time in the United States, isotemporal substitution models have been applied to study the association of sleeping, sedentary behavior, and physical activity with phenotypic age acceleration.

## Key points

1. 30 min/day spent on sedentary behavior was positively associated with PhenoAgeAccel.
2. 30 min/day spent on leisure-time MVPA was adversely associated with PhenoAgeAccel.
3. Replacing 30 min/day sedentary behaviors with 30 min/day of MVPA or 30 min/day of walking/bicycling was adversely associated with PhenoAgeAccel.
4. Substituting 30 min/day of walking/bicycling for 30 min/day of leisure-time MVPA was positively associated with PhenoAgeAccel.

## Welfare of animals

This article does not contain any studies with animals performed by any of the authors.

## Study registration

This study was not formally registered.

## Analytic plan pre-registration

The analysis plan was not formally pre-registered.

## Background

The world's population is growing older due to life expectancy increasing and fertility levels decreasing. By 2050, the world's elderly population will reach one in six [1]. The aging process is associated with an increased risk of many chronic diseases [2, 3]. From the economic perspective, a delay of 2.2 years in aging would save seven trillion dollars over the next 50 years [4]. As time goes on, aging refers to changes in body composition, internal balance, energy, and brain health [5]. According to previous studies, aging may lead to degenerative loss of muscle mass and quality [6], neurodegeneration [7], cardiovascular homeostasis and metabolic disturbances [8], among others, and affects every organ in the body. However, Chronological age (CA) is not a perfect proxy for the true biological aging status of the body [9]. A new biological aging measure, PhenoAgeAccel, has been proved to identify the risk of morbidity and mortality among different subpopulations in the U.S, especially among adults who were healthy and free of diseases [10]. As a complex process, aging also involves the interaction of physiologic and lifestyle factors.

Based on the latest recommendations for Americans' Physical Activity are the following: active adults should do 150 to 300 min of moderate-intensity exercise every week, or 75 min of vigorous-intensity aerobic exercise every week, or an equivalent combination of exercise that is both moderate and vigorous intensity [11]. It is crucial to prevent the aging onset, perpetuation, and progression by enhancing physical activity, especially moderate-to-vigorous intensity [12, 13], which is defined as energy expenditure ≥ 3 metabolic equivalents (METs) [14].On the other hand, some evidence has identified that sedentary behavior is a significant predictive factor of aging [15, 16], with energy expenditure ≤ 1.5 METs [14]. Meanwhile, the research on aging and sleeping duration is scant, and results have been controversial [17–20], with shorter or longer sleeping duration is associated with accelerating cellular aging.

However, most studies [9, 17] failed to reflect the competitive relationship between sleeping, sedentary behavior, and physical activity within a fixed period. The increment of moderate-to-vigorous physical activity (MVPA) for one hour requires a corresponding decrease in one hour of other forms of physical activity, sedentary behavior, or sleeping. It is important to consider such relationships when we analyze the effects of certain types of physical activity on health outcomes. According to the seminal works of Mekary et al. [21], isotemporal substitution model (ISM) was put forward to evaluate the potential effect on health outcomes of substituting one specific type of activity with another [22].

To the best of our knowledge, little is known about the complex inter-relationships of sleeping, sedentary behavior, and physical activity with aging. In order to fill these gaps in the literature, the objective of this study is to use the isotemporal substitution model to explore the relationships between these health-related behaviors and aging. We hypothesized that substituting 30 min of sedentary behaviors with the same amount of sleep, walking/bicycling or MVPA would slow biological aging.

## Methods

### Study population

The National Health and Nutrition Examination Survey (NHANES) is a cross-sectional survey of the US population. A two-year cycle of the NHANES was conducted, and a nationally representative sample was chosen using a multistage sampling design [23]. The survey is conducted annually among a national sample of about 5,000 people. These people are located in counties throughout the country and visit 15 of them each year. NHANES include interviews and physical examinations. NHANES interviews cover demographic, socioeconomic, diet and health-related issues; The latter includes clinical, physiological and laboratory assessments administered by trained medical personnel [24].

From 2007 to 2010, 12,153 participants aged 20 or older participated in the NHANES study. We excluded the participants who lacked PhenoAgeAccel information (*N* = 6842); and those with incomplete physical activity questionnaires (*N* = 23). Finally, 5288 participants were involved in our study (Fig. S1).

### Physical activity measures

Data were derived from questionnaire measurements; The Physical Activity Questionnaire (PAQ) is based on the Global Physical Activity Questionnaire (GPAQ) [25]. The Physical Activity questionnaire included 19 items of physical activity, providing information on walking/ bicycling time, vigorous and moderate-intensity activity, and sedentary activity. Relevant physical activities in the questionnaire included Vigorous work-related activity, Moderate work-related activity, Walking or bicycling for transportation, Vigorous leisure-time physical activity, Moderate leisure-time physical activity. In addition, participants were asked to answer a separate question about how much time they spent sitting each day. For each physical activity, you will be asked if you do it, then how many days a week you do it, and finally how long you do it each day. The number of days calculated was multiplied by the number of hours of exercise per day to determine how much time participants spent on different types of physical activity in a given week.

In this study, moderate leisure-time physical activity and vigorous leisure-time physical activity were combined to form MVPA. MVPA minutes per week was calculated using the formula: [moderate leisure-time activity minutes × moderate leisure-time days] + [vigorous leisure-time activity minutes × vigorous leisure-time days] = mvpa minutes per week. According to MVPA, participants were classified into two categories: MVPA < 150 min/week, MVPA ≥ 150 min/week [11]. [walking/ bicycling minutes × walking/ bicycling days] = walking/ bicycling minutes per week [26].

### Sleeping

Assessments were conducted on sleeping duration during workdays and weekends. In response to the NHANES question: " How much sleep do you usually get at night on weekdays or workdays?" [27]. The sleep duration was categorized as short (< 7 h per night), normal (≥ 7 h per night), and normal used as the reference group.

### Phenotypic age acceleration

PhenoAgeAccel was calculated according to the formula proposed by Levine et al. [28]. It was calculated by age and nine biomarkers including albumin, creatinine, glucose, C-reactive protein, lymphocyte percentage, mean cell volume, red cell distribution width, alkaline phosphatase, white blood cell count (Supplementary methods) [29]. Biomarkers data data were extracted based on “Albumin & Creatinine—Urine, Plasma Fasting Glucose & Insulin, C-Reactive Protein (CRP), Complete Blood Count with 5-part Differential—Whole Blood, Standard Biochemistry Profile” from NHANES Laboratory Data. PhenoAgeAccel is the symbol of phenotypic aging after considering the effects of chronological age [30].

### Other covariates

In this study, we adjusted for sex, ethnicity/race, education, drinking, smoking, coffee, and body mass index (BMI) to control for confounding bias. Table S1 gives details about covariates. Sex, race and education were obtained from demographics data; Information on alcohol, smoking and coffee intake was collected from questionnaire data. To calculate BMI (kg/m^2^), weight (kg) was divided by height squared(m^2^). In addition, overweight was defined by a BMI of above 28 kg/m^2^ [31].

### Statistical analyses

For the description of the baseline characteristics of the sample, we computed means and standard deviation for continuous variables while frequencies were calculated for categorical variables. The associations between each PA and PhenoAgeAccel were estimated using multivariate linear regression analyses. To estimate associations of each activity with PhenoAgeAccel, separate models were used to estimate all exercise intensity levels (sleeping, SB, MVPA, and walking/ bicycling). Each of the covariates mentioned above was adjusted [32].

In an isotemporal substitution model, a defined duration of one physical activity intensity is replaced with the same duration of another physical activity intensity [21, 33]. The regression coefficients of these models describe the distinctions in PhenoAgeAccel with various types of physical activity for 30 min. Statistically significant differences were found in the regression model. Three of the four physical activity variables (sleeping, sedentary, walking/bicycling, MVPA) were continually included in models, and the total time and other covariates were adjusted. Results are presented as β-values and confidence intervals (95% CIs) [34]. There is a significant association between the outcome variable and a covariate if the regression coefficient, beta, is significantly different to zero.

As a result of the complex NHANES sampling design, nonresponse, and oversampling, all analyses were incorporated with interview weights provided by NHANES. Analyses of all statistical tests were conducted using IBM SPSS Statistics 21.0 software (IBM, Asia Analytics Shanghai).

## Results

### Characteristics of participants

A significantly lower PhenoAgeAccel occurred in participants who had MVPA ≥ 150 min/week compared to those who had MVPA < 150 min/week (Table 1). Moreover, participants who had MVPA ≥ 150 min/week engaged in more walking/bicycling, and fewer sleeping.

**Table 1:** The characteristics of the participants were divided by MVPA

Characteristic	MVPA	
	**MVPA < 150 min/week (*n* = 3845)**	**MVPA ≥ 150 min/week (*n* = 1443)**
**Weighted frequency**	143,288,974	70,118,799
**Sex, %**		
** Male**	1737(45.1)	807(55.9)
** Female**	2108(54.8)	636(44.0)
**Ethnicity/race, %**		
** Non-Hispanic White**	1763(45.8)	759(52.5)
** Others**	2082(54.1)	684(47.4)
**Education, %**		
** Under high school**	1373(35.7)	212(14.6)
** High school or above**	2464(64.0)	1230(85.2)
**BMI (kg/m**^**2**^**)**		
** Under & health weight**	1015(26.3)	498(34.5)
** Overweight**	2764(71.8)	938(65.0)
**Smoking status, %**		
** Non-smoker**	2012(52.3)	839(58.1)
** Ever-smoker**	943(24.5)	374(25.9)
** Current-smoker**	887(23.0)	230(15.9)
**Drinking, %**		
** Non-drinker**	528(13.7)	124(8.5)
** Ever-drinker**	554(14.4)	137(9.4)
** Current-drinker**	2406(62.5)	1064(73.7)
**Coffee, %**		
** < 1 cup/day**	2723(70.8)	1061(73.5)
** ≥ 1 cup/day**	482(12.5)	183(12.6)
**Sleeping, 30 min/day**	13.63 ± 3.06	13.60 ± 2.61
**Sedentary behavior, 30 min/day**	10.53 ± 6.78	10.57 ± 6.51
**MVPA, 30 min/day**	0.09 ± 0.18	1.95 ± 1.77
**Walking/bicycling, 30 min/day**	0.40 ± 1.45	0.50 ± 1.41
**PhenoAgeAccel**	-3.88 ± 8.34	-6.17 ± 6.80

Values were means ± SD or n (percentages)

*BMI* Body mass index, *MVPA* Moderate-to-vigorous physical activity, *PhenoAgeAccel* Phenotypic age acceleration

### Independent and partition models

The association of 30 min/day of sleeping, sedentary behavior, walking/bicycling, and MVPA with PhenoAgeAccel was depicted in Table 2. As shown in the total participants, 30 min/day of SB was positively related to PhenoAgeAccel (*β* = 0.07, 95% CI: 0.04, 0.11), while 30 min/day of MVPA for leisure time was adversely related to PhenoAgeAccel (*β* =  − 0.55, 95% CI: − 0.73, − 0.38). After multivariate adjustments (Model 2 and Model 3), the above two associations remained (*β* = 0.07, 95% CI: 0.05, 0.10; *β* =  − 0.35, 95% CI: − 0.51, − 0.19). In this study, the score for MET from walking or bicycling was close that of moderate leisure-time physical activity, resulting in MVPA results similar to those of walking or bicycling.

**Table 2:** Associations (*β*(95% CI)) of 30 min/day of sleeping, sedentary behavior, walking/bicycling and MVPA with PhenoAgeAccel among adults

Variables	Model 1	Model 2	Model 3
Sleeping	**-0.10(-0.19, -0.02)**	-0.03(-0.10, 0.05)	-0.03(-0.11, 0.04)
Sedentary behavior	**0.07(0.04, 0.11)**	**0.08(0.05, 0.11)**	**0.07(0.05, 0.10)**
Walking/bicycling	**-0.11(-0.24, -0.01)**	**-0.14(-0.25, -0.03)**	-0.09(-0.21, 0.03)
MVPA	**-0.55(-0.73, -0.38)**	**-0.39(-0.55, -0.22)**	**-0.35(-0.51, -0.19)**

Model 1 Adjusted for none

Model 2 Adjusted for sex, ethnicity/race, education, smoking, body mass index

Model 3 Adjusted for sex, ethnicity/race, education, smoking, body mass index, additionally adjusted for the other three variables of activity status

*MVPA* Moderate-to-vigorous physical activity, *CI* Confidence intervals

Associations analysis of PhenoAgeAccel with sleeping, sedentary behavior, walking/bicycling, and MVPA of the study population stratified to the underlying MVPA are depicted in Table 3.

**Table 3:** Associations analysis of (*β*(95% CI)) PhenoAgeAccel with sleeping, sedentary behavior, walking/bicycling and MVPA in different MVPA populations

Variables	MVPA < 150 min/week			MVPA ≥ 150 min/week		
	Model 1	Model 2	Model 3	Model 1	Model 2	Model 3
Sleeping	**-0.09(-0.21, -0.03)**	-0.03(-0.13, 0.07)	-0.04(-0.14, 0.06)	**-0.11(-0.20, -0.02)**	-0.01(-0.13, 0.12)	-0.01(-0.13, 0.12)
Sedentary behavior	**0.11(0.07, 0.15)**	**0.11(0.08, 0.13)**	**0.10(0.08, 0.13)**	0.002(-0.06, 0.06)	0.01(-0.05, 0.07)	0.01(-0.05, 0.07)
Walking/bicycling	**-0.21(-0.34, -0.08)**	**-0.22(-0.35, -0.10)**	**-0.21(-0.34, -0.08)**	0.21(-0.04, 0.46)	0.15(-0.09, 0.39)	0.16(-0.08, 0.41)
MVPA	**-4.82(-6.03, -3.60)**	**-3.15(-4.40, -1.91)**	**-3.29(-4.58, -2.00)**	0.04(-0.15, 0.23)	-0.02(-0.21, 0.16)	-0.04(-0.23, 0.14)

Model 1 Adjusted for none

Model 2 Adjusted for sex, ethnicity/race, education, smoking, body mass index

Model 3 Adjusted for sex, ethnicity/race, education, smoking, body mass index, additionally adjusted for the other three variables of activity status

*MVPA* Moderate-to-vigorous physical activity, *CI* Confidence intervals

### Isotemporal substitution models

The ISM analysis of the association of 30 min/day spent on sleeping, walking/bicycling, sedentary behaviors, and MVPA with PhenoAgeAccel was presented in Table 4. After adjusting all of the confounders, replacing 30 min/day spent on MVPA for leisure time with an equal amount of sleeping was positively related to PhenoAgeAccel (*β* = 0.32, 95% CI: 0.16, 0.48). Moreover, replacing 30 min/day spent on MVPA for leisure time with an equal amount of sedentary behaviors was positively related to PhenoAgeAccel (*β* = 0.43, 95% CI: 0.26, 0.60). In contrast, replacing 30 min/day spent on sedentary behavior with an equal amount MVPA for leisure time was adversely related to PhenoAgeAccel (*β* = -0.43, 95% CI: -0.60, -0.26); replacing 30 min/day spent on sleeping with equal amount leisure time MVPA was adversely related to PhenoAgeAccel (*β* = -0.32, 95% CI: -0.48, -0.16).

**Table 4:** Isotemporal substitution model of associations (*β*(95% CI)) of 30 min/day of sleeping, sedentary behavior, walking/bicycling and MVPA with PhenoAgeAccel among adults

	Sleeping	Sedentary behavior	Walking/bicycling	MVPA
Overall				
Replacing sleep	Dropped	**0.11(0.04, 0.18)**	-0.05(-0.19, 0.08)	**-0.32(-0.48, -0.16)**
Replacing sedentary	**-0.11(-0.18, -0.04)**	Dropped	**-0.16(-0.29, -0.04)**	**-0.43(-0.60, -0.26)**
Replacing walking	0.05(-0.08, 0.19)	**0.16(0.04, 0.29)**	Dropped	**-0.27(-0.49, -0.04)**
Replacing MVPA	**0.32(0.16, 0.48)**	**0.43(0.26, 0.60)**	**0.27(0.04, 0.49)**	Dropped
MVPA < 150 min/week				
Replacing sleep	Dropped	0.06(-0.10, 0.22)	**-0.83(-1.04, -0.62)**	**-3.92(-6.12, -1.72)**
Replacing sedentary	-0.06(-0.22, 0.10)	Dropped	**-0.89(-1.10, -0.68)**	**-3.98(-6.22, -1.74)**
Replacing walking	**0.83(0.62, 1.04)**	**0.89(0.68, 1.10)**	Dropped	**-3.09(-5.25, -0.93)**
Replacing MVPA	**3.92(1.72, 6.12)**	**3.98(1.74, 6.22)**	**3.09(0.93, 5.25)**	Dropped
MVPA ≥ 150 min/week				
Replacing sleep	Dropped	0.01(-0.27, 0.29)	0.26(-0.13, 0.64)	0.004(-0.31, 0.32)
Replacing sedentary	-0.01(-0.29, 0.27)	Dropped	0.25(-0.19, 0.69)	-0.01(-0.34, 0.33)
Replacing walking	-0.26(-0.64, 0.13)	-0.25(-0.69, 0.19)	Dropped	-0.25(-0.76, 0.26)
Replacing MVPA	-0.004(-0.32, 0.31)	0.01(-0.33, 0.34)	0.25(-0.26, 0.76)	Dropped

Adjusted for sex,ethnicity/race, education, smoking, body mass index, additionally adjusted for the other three variables of activity status

*MVPA* Moderate-to-vigorous physical activity, *CI* Confidence intervals

In this association, 30 min/day spent on SB was replaced by an equal amount MVPA (*β* = -3.98, 95% CI: -6.22, -1.74) or walking/bicycling (*β* = -0.89, 95% CI: -1.10, -0.68) was adversely related to PhenoAgeAccel; replacing 30 min/day spent on walking/cycling with equal amount leisure time MVPA was positively related to PhenoAgeAccel (*β* = 3.09, 95% CI: 0.93, 5.25), still existed among those MVPA < 150 min/week. In the population of MVPA ≥ 150 min/week, no such association has been found (Table 4).

## Discussion

Our work provided evidence that walking/bicycling, sleeping, and the MVPA for leisure time were adversely related to PhenoAgeAccel. In contrast, sedentary behavior was positively associated with PhenoAgeAccel. Replacing SB with an equal amount of bicycling/walking, sleeping, or MVPA or substituting bicycling/walking with MVPA was adversely related to PhenoAgeAccel, particularly among those with no more than 150 min MVPA per week [35, 36]. Based on the sample size for most adults with MVPA < 150 min/week, perhaps the low PA group is driving the analysis for associations between sedentary, walking/bicycling, and MVPA.

According to the studies of Chunyu Xin et al., they discovered that the long nighttime sleep duration (≥ 8 h/night) was associated with a lower likelihood of aging [37]. Yi-Hsuan Lin et al.’s study reported that physically active middle-aged and older adults were less likely to aging than sedentary adults [38]. Based on a sample of nationally representative adults and modeling isotemporal substitution with consideration of potential factors, our study on the relationship of PA with aging increased the validity and relevance of the evidence [39]. The modeling isotemporal substitution allows comparing normative exchanges of a fixed amount of one activity for the same amount of another PA based on empirically-derived data [21]. ISM shows the accuracy enhanced estimate of the relationships of physical activities and sedentary behaviors with PhenoAgeAccel compared to the conventional modeling [39]. Considering that a day has a finite amount of time and the intensity of physical activity, the isotemporal substitution modeling also estimates the effects of changing activity for another [24]. In epidemiological studies of physical activity, the ISM has been broadly used to replace PA effects with health. According to the studies of Martins et al. [40], they discovered that replacing the time spent sitting or sleeping with the same amount of MPA time may reduce frailty. It has been shown in these studies that physical activity, especially MVPA, can lead to positive health outcomes when replaced with time for sedentary behaviors [41]. Compared with studies without consideration of the nature of competition, a more solid basis for evidence can be found using isotemporal substitution models [29].

Based on a sample of large nationally representative adults, physical activity may be able to reduce the burden of aging in America, according to the results of our study [39]. In our study, physical activity is associated with a lower PhenoAgeAccel, and the benefits of MVPA are greater than bicycling/walking and sleeping. Modeling of isotemporal substitutions is scarcely supported by evidence on PhenoAgeAccel compared to other health outcomes among adults. Research by Saunders found that health benefits were most consistently associated with high levels of physical activity, especially MVPA [30]. The study from Sun et al. also found a preliminary connection between poor sleeping and frailty, one of the syndromes associated with aging [42]. PhenoAgeAccel represents an aging process that affects multiple systems characterized by complex biological mechanisms, though what we found complements the result. Many studies have used isotemporal models showing the replacement of lower-intensity behaviors with higher-intensity behaviors results in better health and lower mortality. In comparison, two isotemporal substitution studies suggested that engaging in a light physical activity instead of sedentary behavior was not projected to result in any benefits for either well-being indicator [27, 32]. Han et al.’s study reported that the number of sleeping hours per day over 8 is positively correlated with an increased PhenoAgeAccel [43]. Participants in different studies may have different characteristics (e.g., country, race/ethnicity, lifestyle), which may account for the different findings [29].

According to our analysis, especially for participants with MVPA less than 150 min, walking/bicycling and sleeping displayed a trend of lower aging compared to sedentary behavior. Physical activity may play an essential role in improving adults' aging and preventing subsequent adverse health outcomes [29, 33]. As a result of the considerable burden on public health brought by aging in the present study, the cost-effectiveness of actively participating in sports, especially MVPA, should be considered to prevent aging [39, 44]. In longitudinal research, physical activities have been related to a lower burden of aging among adults, and future studies will likely consider the competing nature of variables related to physical activity [34]. In addition to MVPA, bicycling/walking and sleeping also contribute to aging.

Multiple underlying mechanisms may account for the benefits on PhenoAgeAccel from physical activity. Regular physical activity can prolong the average human life span by affecting the development of chronic diseases, alleviating aging degeneration and its impact on health, and maintaining physical function. PA promotes health by allocating energy away from potentially harmful overinvestments in fat storage and reproductive tissues and PA also stimulates energy allocation toward repair and maintenance processes [45]. Physical activity also increases longevity and survival. For middle-aged and older people, a dose–response relationship was found between physical activity and decrease in mortality. Compared with sedentary older people, physically active older adults were more likely to remain living independently. Physical activity in old age preserves the cognitive and physical functions [38].

One major limitation of our study is that the cross-sectional study design cannot make causal relationship inference [29]. The temporal relationships between sleeping, sedentary behavior, physical activity, and PhenoAgeAccel cannot be determined. And future longitudinal studies are still needed to better explore the causal relationship between different variables. Another limitation involves measuring physical activity: there is a possibility that GPAQ data may not be as accurate as accelerometer data, although GPAQ has been validated in various populations [46]. Different intensities (with METS < 4) of physical activity data were not collected by the GPAQ [39]. In addition, despite including many covariates, confounders that are unmeasured and residual may still exist. Moreover, a variety of lifestyle information was reported by themselves; therefore, recall bias may exist [47]. Despite these limitations, PhenoAgeAccel is a marker that may be used to monitor the aging process before symptoms of diseases appear [10]. A comprehensive quality control and quality assurance procedure are applied to NHANES to ensure that data is accurate and reliable. Furthermore, this study has a sample of nationally representative adults in America, and many samples were used, making the results more generalizable [29]. Therefore, in terms of public health, the findings have more significance regarding disease prevention.

## Conclusions

In conclusion, replacing SB with an equal amount of bicycling/walking, sleeping, or MVPA for leisure time is adversely related to PhenoAgeAccel in adults. The findings of our study emphasize the significance of adhering to physical activity, especially for adults exposed to MVPA, for no more than 150 min per week. It is necessary for future research on the relationship between sedentary behavior and PA with aging, considering the nature of competition and examining physical activity and its effectiveness in preventing aging.

## Supplementary Information

**Additional file 1: Supplementary Methods. Figure S1.** Flow chart of the population included in the final analysis of our study. **Table S1.** The Classifications of Covariates.

## Data Availability

The data supporting this study’s findings are available at https://www.cdc.gov/nchs/nhanes/about_nhanes.htm. Information from NHANES is made available through extensive publications and articles in scientific and technical journals. For data users and researchers worldwide, survey data and easy-to-use CD-ROMs are available on the internet.

## Abbreviations

BMI: Body mass index
CI: Confidence interval
GPAQ: Global physical activity questionnaire
ISM: Isotemporal substitution model
METS: Metabolism equivalents
MVPA: Moderate-to-vigorous physical activity
NHANES: National Health and Nutrition Examination Survey
PAQ: Physical activity questionnaire
PhenoAgeAccel: Phenotypic age acceleration
SB: Sedentary behavior
