# Supplementary material for: Associations of sleeping, sedentary and physical activity with phenotypic age acceleration: a cross-sectional isotemporal substitution model
Source: BMC Geriatr. 2023 Mar 23;23:165. doi: 10.1186/s12877-023-03874-6 (PMC10035275; doi:10.1186/s12877-023-03874-6)
Supplement: Supplementary file 1 — Additional file 1: Supplementary Methods. Figure S1. Flow chart of the population included in the final analysis of our study. Table S1. The Classifications of Covariates. [file 12877_2023_3874_MOESM1_ESM.docx]

**Supplementary Materials**

**Associations of sleeping, sedentary and physical activity with phenotypic age acceleration:** **a cross-sectional isotemporal substitution model**

**Appendix 1: Supplementary Methods**

**Appendix 2: Supplemental Results**

**Appendix 1**

**Supplementary Methods**

**2.1 Study population**

In this study, we used data from two NHANES survey cycles (2007–2008 and 2009–2010) to investigate sleeping, sedentary behavior, physical activity and phenotypic age acceleration.

**2.2 Measurements and variables**

PhenoAgeAccel was calculated by age and nine biomarkerswhich were shown below:

Phenotypic Age = 141.50 + $\frac{\ln\left\{ -0.00553\times\ln\left( 1-mortality risk \right) \right\}}{0.090165}$

where Mortality risk = 1 − exp ($\frac{-1.51714 \times exp (xb)}{0.0076927}$)

and

xb = − 19.907 − 0.0336 × albumin + 0.0095 × creatinine +0.1953 × glucose +

0.0954 × *ln* (C−reactive protein) − 0.0120 × lymphocyte percentage + 0.0268 × mean cell volume + 0.3306 ×red blood cell distribution width + 0.00188 × alkaline phosphatase +0.0554 × white blood cell count + 0.0804 × chronological age

PhenoAgeAccel = Phenotypic Age − Age

**Appendix 2**

**Supplementary Results**

**
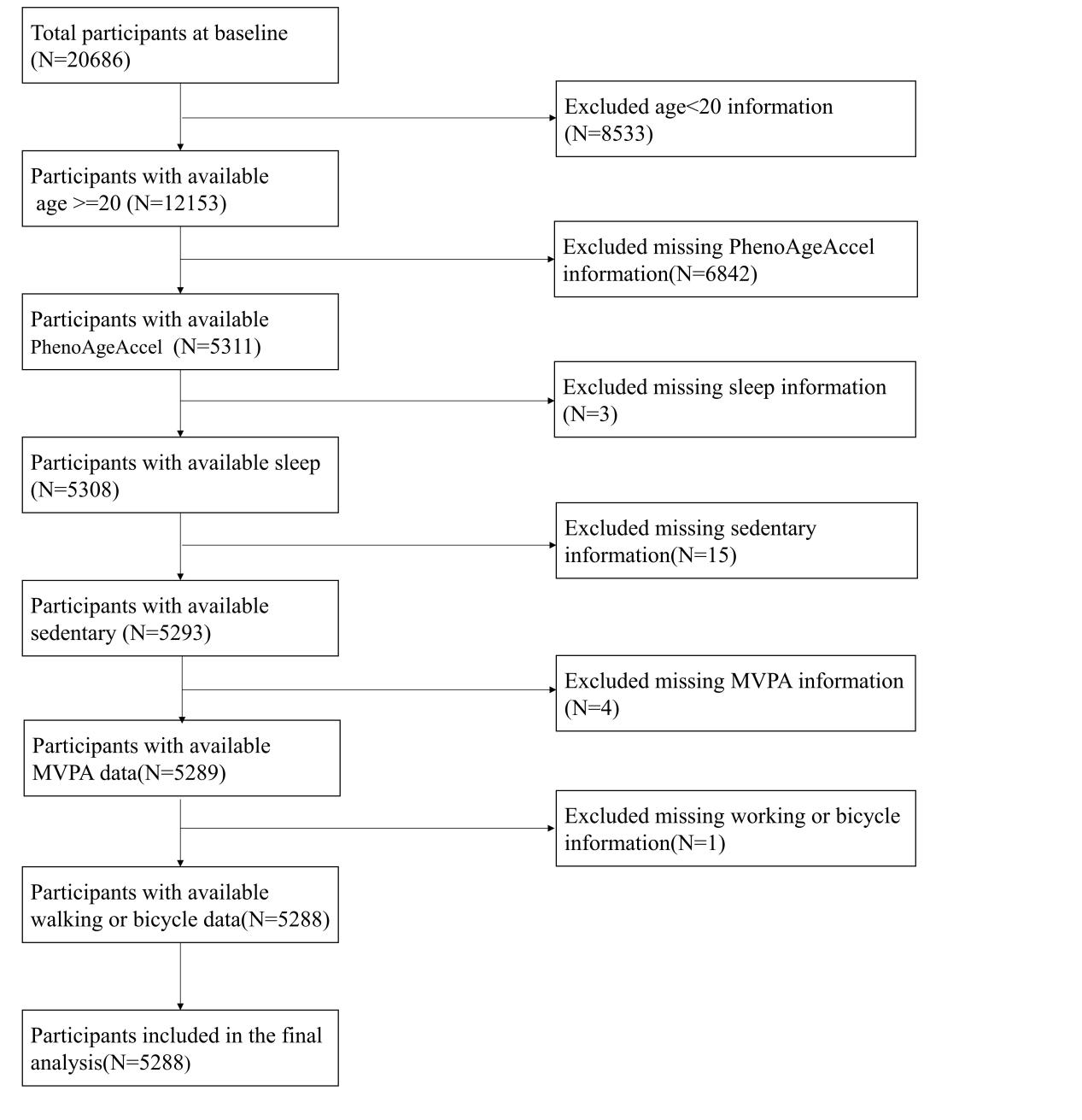
**

Figure S1 Flow chart of the population included in the final analysis of our study.

Table S1. The Classifications of Covariates.

| **Covariates** | **Classifications** |
| --- | --- |
| Sex | 1: Men; 2: women |
| Race | 0: non-Hispanic white; 1: others |
| Education | 0: under high school; 1: high school or above |
| Drinking | 0: non-drinker; 1: ever-drinker; 2: current-drinker |
| Smoking status | 0: non-smoker; 1: ever-smoker; 2: current-smoker |
| Coffee | 0: <one cup per day; 1: ≥one cup per day) |
| Body mass index | 0: ≤28 kg/m^2^; 1: > 28 kg/m^2^ |
